# Supplementary material for: A flexible multi-metric Bayesian framework for decision-making in Phase II multi-arm multi-stage studies
Source: Stat Med. Author manuscript; Available in PMC 2025 Feb 24. (PMC7617374; doi:10.1002/sim.9961)
Supplement: Supplementary Material [file EMS202887-supplement-Supplementary_Material.pdf]

A Flexible Multi-Metric Bayesian Framework for Decision-Making in Phase II Multi-Arm Multi-Stage Studies

Supplemental Material

Suzanne M. Dufault, Angela M. Crook, Katie Rolfe, Patrick P.J. Phillips

September 2023

A Supplemental Material

A.1 Data-generating parameter values

TTP data was generated using the following values for the parameters specified in Eq. 1, with coefficients $\beta_2, \beta_3, \beta_4, \beta_5$ specified according to the scheme defined in Table 2.

$$
\begin{aligned}
\beta_0 &= 0.860 \\
\beta_1 &= 0.083 \\
\sigma_{g_1} &= 0.125 \\
\sigma_{g_2} &= 0.030 \\
\sigma_e &= 0.206 \\
\rho &- 0.317
\end{aligned}
$$

A.2 Bayesian multilevel model

The notation used here follows that described in 2.3.2. The outcome TTP is measured for each individual i at time j and (once log-transformed) is assumed to be normally distributed with mean μ_{ij} and variance σ_y^2. The mean is allowed to differ by arm $X_i = k$, $k = 1, \ldots, K$, where $k = 1$ is the control arm. Arm assignment is assumed to be fixed for all time j for each individual i.

$$
\begin{aligned}
\log_{10}(\mathrm{TTP}_{ij}) &\sim N(\mu_{ij}, \sigma_y^2) \\
\mu_{ij} &= \beta_{0i} + \beta_{1i} \cdot T_{ij} + \beta_2 \mathbb{I}\{X_i = 2\} \cdot T_{ij} + \cdots + \beta_K \mathbb{I}\{X_i = K\} \cdot T_{ij} \\
\begin{pmatrix} \beta_{0i} \\ \beta_{1i} \end{pmatrix} &\sim N\left(\begin{pmatrix} \beta_0 \\ \beta_1 \end{pmatrix}, \Sigma \right), \qquad \Sigma = \begin{pmatrix} \sigma_0^2 & \rho\sigma_0\sigma_1 \\ \rho\sigma_0\sigma_1 & \sigma_1^2 \end{pmatrix} \\
\beta_k &\sim N(0, 2^2), \qquad \forall k = 0, 1, \ldots, K \\
\sigma_y &\sim \text{half Student-t}(df = 3, \mu = 1.2, \sigma = 2.5) \\
\sigma_g &\sim \text{half Student-t}(df = 3, \mu = 0, \sigma = 2.5) \qquad \forall g = 0, 1
\end{aligned}
\tag{A.1}
$$

The default priors from the `brms` package were used to set the priors on the group- and population-level standard deviation,[1, 2] which are based on guidance from [3].

A.3 Simulation conditions

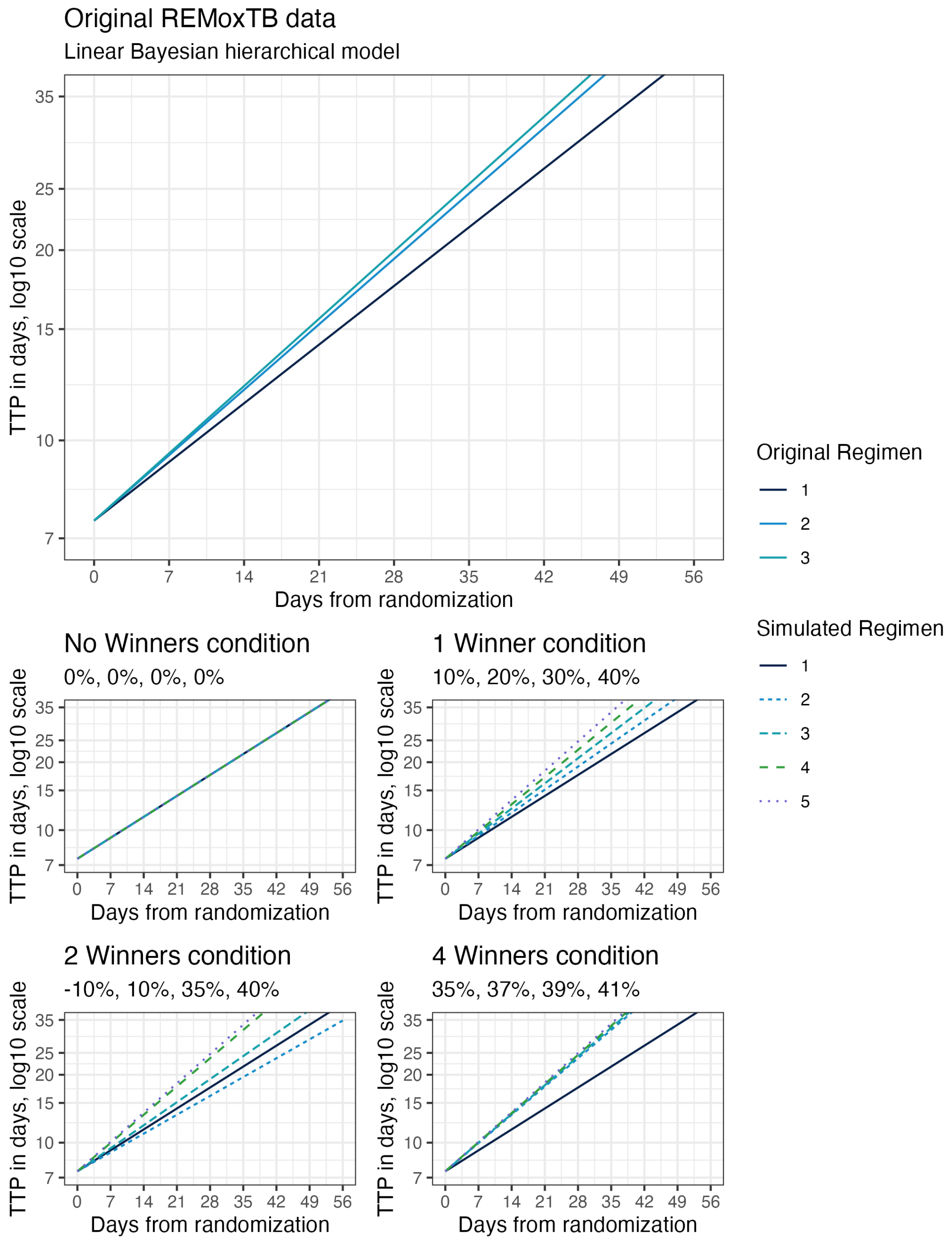

Figure A.1: Simulation settings for time to positivity.

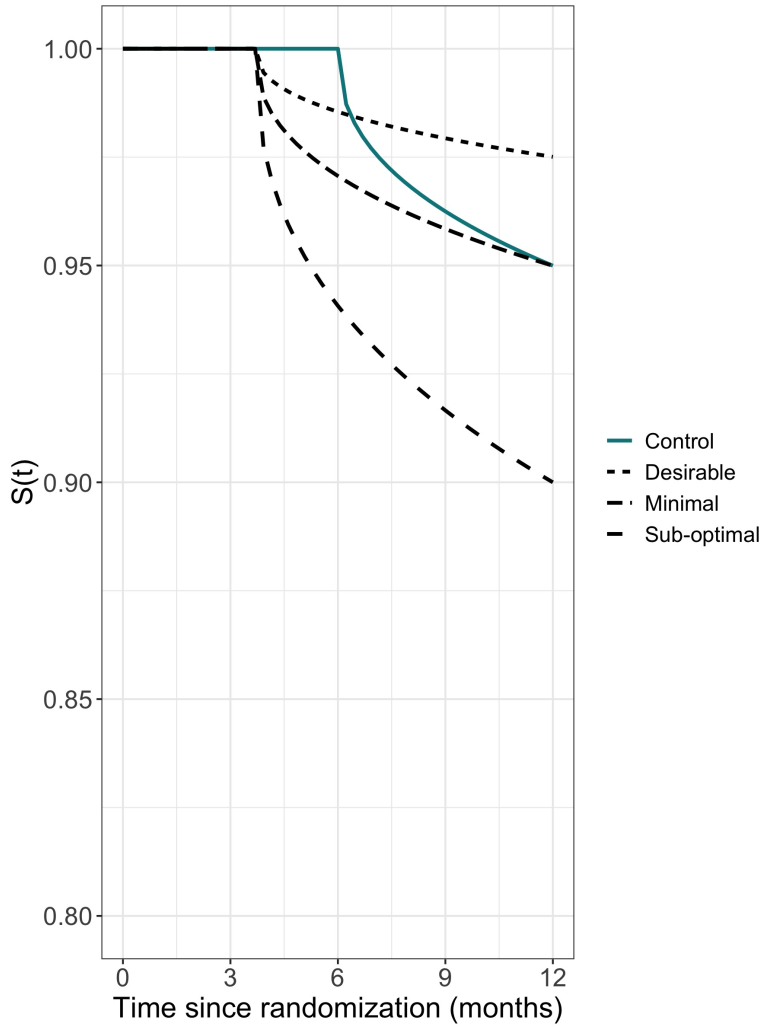

Figure A.2: Simulation settings for unfavourable event rates.

A.4 Evaluation of the proposed metrics as an overall package, alternate settings

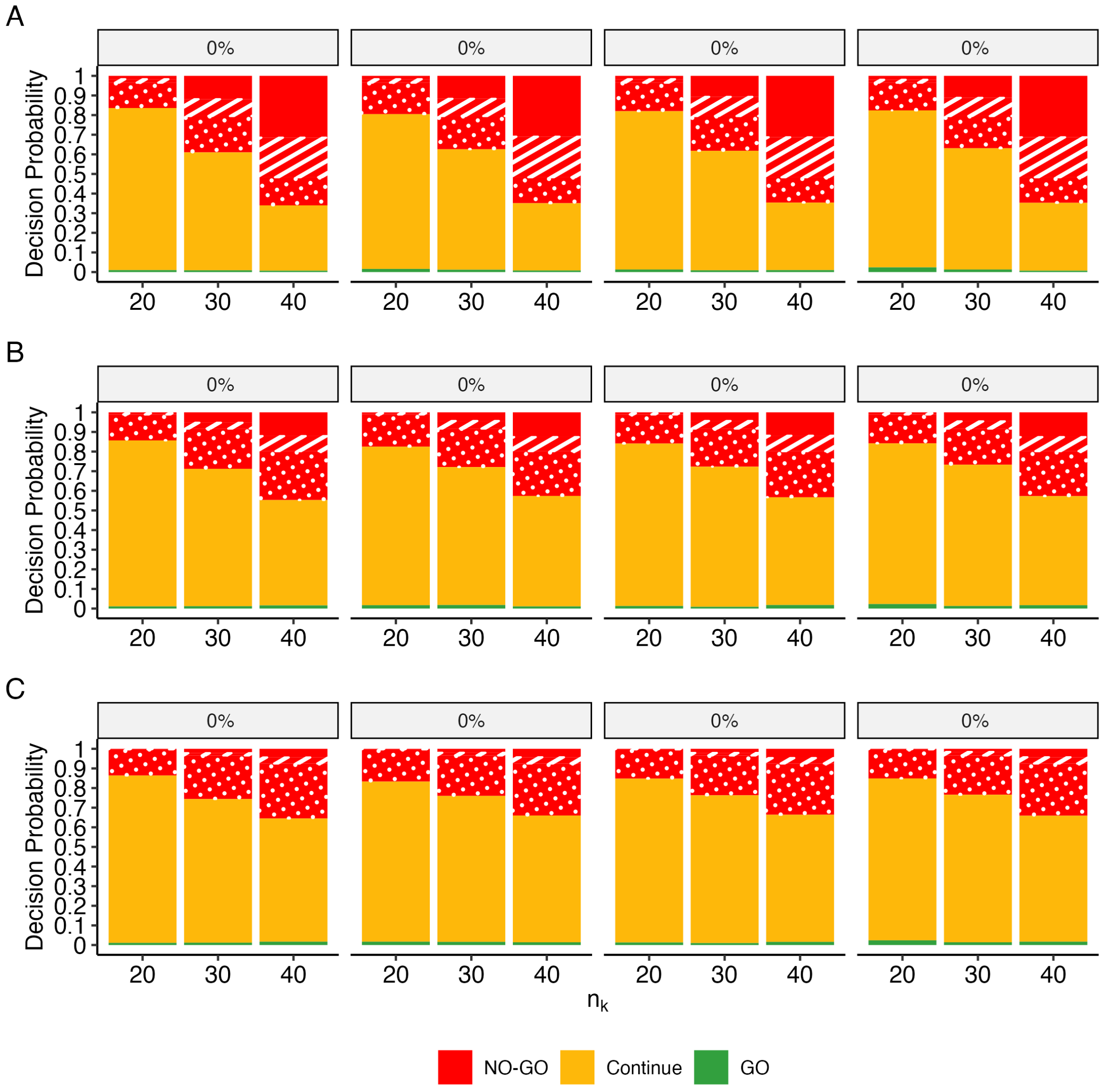

Figure A.3: Interim analysis decision probabilities for simulated arms with TTP slope relative to the control as specified in the panels (all corresponding to the '0 Winners' simulation setting) and unfavorable outcome rates of A) 10% (unfavorable), B) 5% (minimal), and C) 2.5% (desirable). Arms are flagged for a GO decision if they meet all the following conditions: fewer than 2 unfavorable outcomes, evidence of meeting the target product profile, and a posterior probability greater than 50% of ranking in the top two arms. A pattern is applied to differentiate the criterion responsible for NO-GO decisions. Arms are flagged for a NO-GO decision if they experience any of the following conditions: 2 or more unfavorable outcomes (stripes), do not have evidence of meeting the target product profile (dots), or meet both conditions (plain). All arms that do not meet the criteria for a GO or NO-GO decision, receive a "Continue" designation. Results are based on 1,000 simulated datasets per setting.

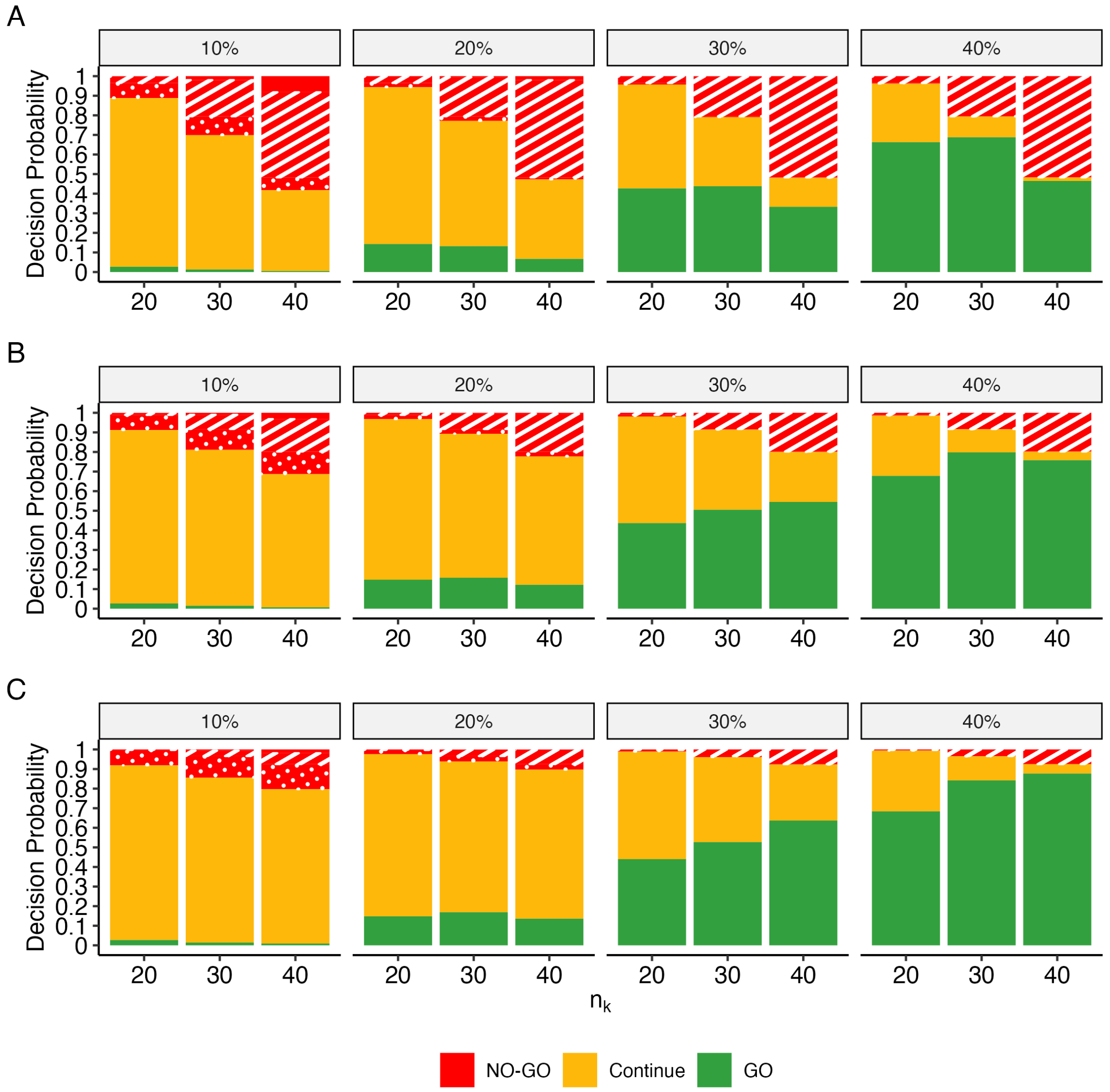

Figure A.4: Interim analysis decision probabilities for simulated arms with TTP slope relative to the control as specified in the panels (all corresponding to the '1 Winner' simulation setting) and unfavorable outcome rates of A) 10% (unfavorable), B) 5% (minimal), and C) 2.5% (desirable). Arms are flagged for a GO decision if they meet all the following conditions: fewer than 2 unfavorable outcomes, evidence of meeting the target product profile, and a posterior probability greater than 50% of ranking in the top two arms. A pattern is applied to differentiate the criterion responsible for NO-GO decisions. Arms are flagged for a NO-GO decision if they experience any of the following conditions: 2 or more unfavorable outcomes (stripes), do not have evidence of meeting the target product profile (dots), or meet both conditions (plain). All arms that do not meet the criteria for a GO or NO-GO decision, receive a "Continue" designation. Results are based on 1,000 simulated datasets per setting.

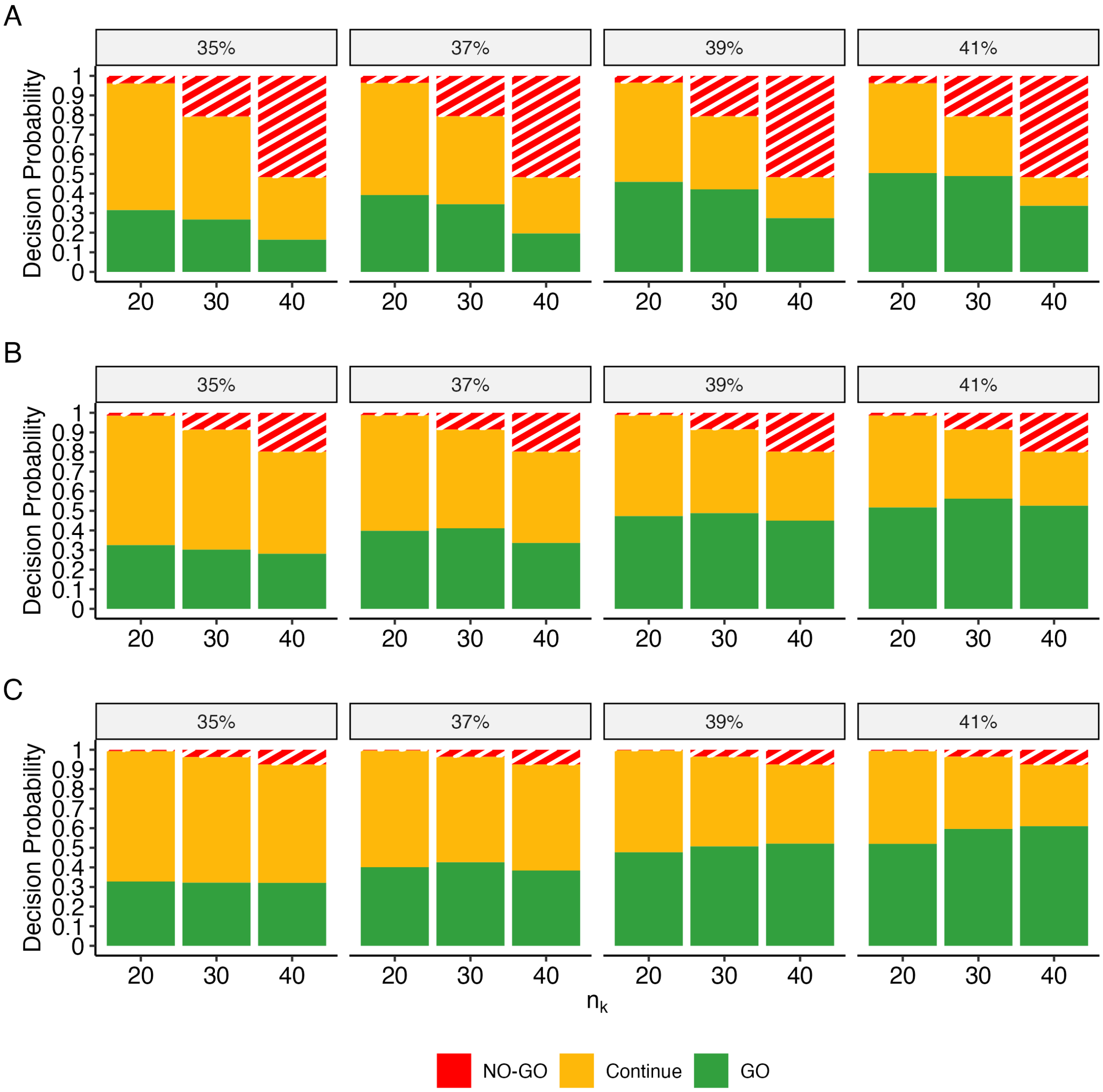

Figure A.5: Interim analysis decision probabilities for simulated arms with TTP slope relative to the control as specified in the panels (all corresponding to the '4 Winners' simulation setting) and unfavorable outcome rates of A) 10% (unfavorable), B) 5% (minimal), and C) 2.5% (desirable). Arms are flagged for a GO decision if they meet all the following conditions: fewer than 2 unfavorable outcomes, evidence of meeting the target product profile, and a posterior probability greater than 50% of ranking in the top two arms. A pattern is applied to differentiate the criterion responsible for NO-GO decisions. Arms are flagged for a NO-GO decision if they experience any of the following conditions: 2 or more unfavorable outcomes (stripes), do not have evidence of meeting the target product profile (dots), or meet both conditions (plain). All arms that do not meet the criteria for a GO or NO-GO decision, receive a "Continue" designation. Results are based on 1,000 simulated datasets per setting.

A.5 Ranking: Time to Positivity Targets

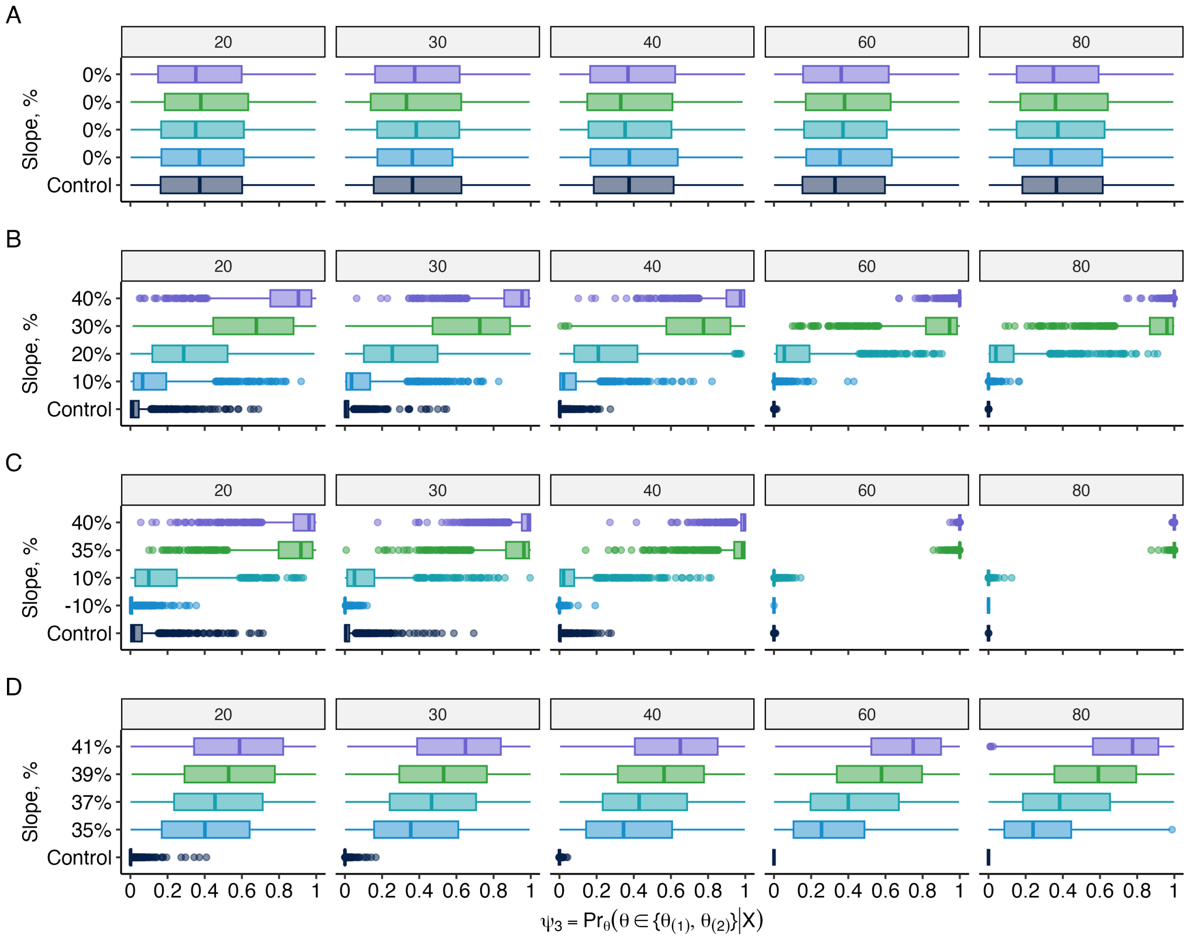

Figure A.6: The estimated posterior probability that a given arm has one of the top two steepest slopes, $\Pr_\theta(\theta_k \in \{\theta_{(1)}, \theta_{(2)}\}|X)$, across varying sample sizes (panels) for the three TTP conditions evaluated: **A)** 'No Winners', **B)**'One Winner', **C)** '2 Winners', **D)** '4 Winners'. Results are based on 1,000 simulated datasets for each sample size and condition.

References

[1] Paul-Christian Bürkner. "Advanced Bayesian multilevel modeling with the R package brms". In: *The R Journal* 10.1 (2018), pp. 395–411. DOI: 10.32614/RJ-2018-017.

[2] Paul-Christian Bürkner. "brms: An R package for Bayesian multilevel models using Stan". In: *Journal of Statistical Software* 80.1 (2017), pp. 1–28. DOI: 10.18637/jss.v080.i01.

[3] Andrew Gelman. "Prior distributions for variance parameters in hierarchical models (comment on article by Browne and Draper)". In: *Bayesian Analysis* 1.3 (2006), pp. 515–534.

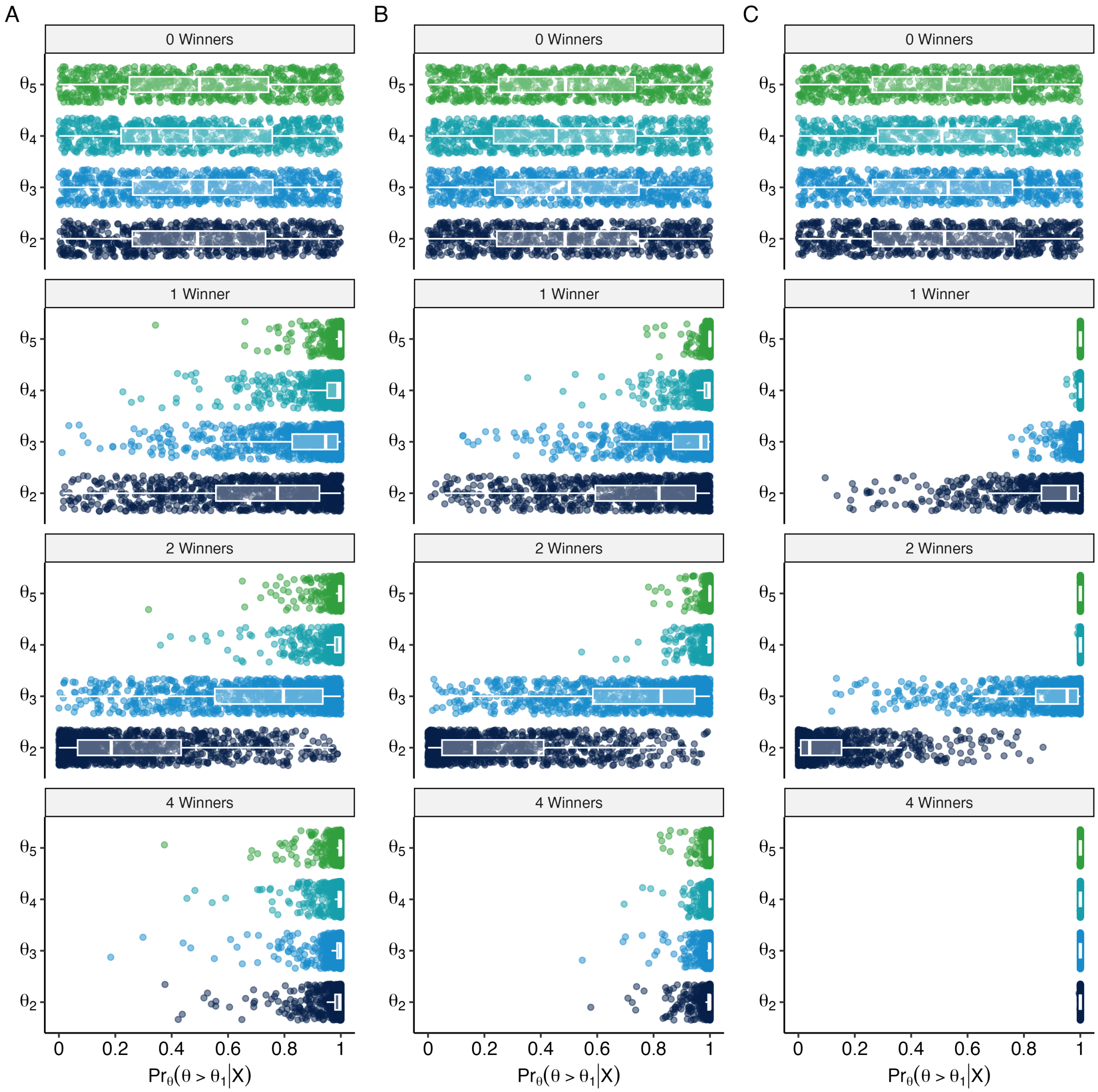

Figure A.7: The estimated posterior probability that a given arm has a steeper slope relative to the control, $\Pr_\theta(\theta_k > \theta_1|X)$, for sample sizes of **A**) 30, **B**) 40, and **C**) 60 per arm.
